# Syphilitic Infection by the Semen

**Published:** 1876-02

**Authors:** 


Syphilitic Infection by the Semen.—In the year 1867, X. contracted six sores upon his glans penis, which he was informed were chancroids by his surgeon (not the writer). These, he says, were readily healed, and have left no visible evidence of their existence. Last January he first applied to me for the treatment of a general feeling of malaise, and I at once suspected syphilis, knowing that his habits had been loose; his countenance was of a pale, dirty, yellowish tinge. I put him, however, simply upon a tonic of iron and gentian, and he immediately felt better. In about two weeks a syphilitic eruption began to show beneath the epidermis, and I gave him the mixed treatment, which held the eruption from further development and finally dispelled it; but there followed mucous patches in the mouth, fauces and nasal passages. He is positive that he has not had an abrasion, or any disease on the penis since the year 1867; at present he is apparently cured.

As soon as he came under my care I warned him very particularly about the chances of communicating his disease to his wife, telling him that his semen might infect her. He said he should use every precaution, but he failed to do so on one occasion, and just four weeks subsequently I found her with four chancres upon the labia minora, and, from the pain produced at the internal os by the application of the uterine sound I judged of the presence there of another chancre. Secondary symptoms followed in her case, with mucous patches of mouth and nares, which have yielded to a mixed treatment. I shall make no comments upon the case. The above are the facts, and the result, I think, has borne out the diagnosis.—*Smith in Archives of Dermatology.*

